# Cerebral oximetry and laparoscopic surgery

**DOI:** 10.4103/0972-9941.26644

**Published:** 2006-06

**Authors:** Eleni Moka

**Affiliations:** Creta Interclinic Hospital, 63, Minoos Street, Heraklion Crete, PC 71304, Greece

Laparoscopic surgery has greatly developed recently and is becoming a standard operative procedure because of the important advantages it offers, namely less tissue trauma, decreased postoperative pain, as well as shorter hospital stay with minimized postoperative morbidity and mortality. It offers patients major medical and economical advantages over laparotomy.

Laparoscopic surgery, although enjoying ever-increasing popularity and presenting new anaesthetic challenges, can be potentially hazardous due to the intraperitoneal insufflation of CO_2_ that is required for the procedure. Laparoscopy may lead to possibly harmful cardiovascular and respiratory alterations that could possibly be harmful for the patient. Numerous studies have focused on the effects of intraperitoneal CO_2_ insufflation and its systemic absorption on cardiovascular, respiratory and on the derangement of the acid-base balance, all of which can potentially impair the cerebral perfusion.

The normal cerebrovascular response to CO_2_ alterations (CO_2_ reactivity) has been analyzed profoundly. Nevertheless, there are only few references regarding the alterations in the cerebrovascular dynamics after CO_2_ insufflation into peritoneal cavity during laparoscopic surgery and the effect of such insufflation on cerebral blood flow velocities (CBFV) and cerebral oxygenation (rSO2). It has been reported that CO_2_ insufflation can produce adverse cerebrovascular effects, although this has not been extensively studied. Cerebral blood flow (CBF) increases during pneumoperitoneum in head-up or head-down positions due to the rise of arterial CO_2_ partial pressure (PaCO_2_). This increase in the CBF could be dangerous if laparoscopy is performed in patients with cerebral disease, reduced intracranial compliance or impaired cerebral physiology.

It is well known that anaesthetic agents have variable effects on cerebral circulation. Consequently, the choice of the anaesthetic regimen could be important in laparoscopic surgery in order to minimize the adverse cerebrovascular response. Cerebral autoregulation mechanism and CO_2_ reactivity, two homeostatic mechanisms important for the control of CBF, are impaired by pathology and some drugs commonly used during anaesthesia.

Regarding the type of anaesthetic agent used, it is already known that inhalation anaesthesia's effects on CBF depend on the balance between its indirect vasoconstrictive action, secondary to flow metabolism coupling and its direct vasodilatory properties. CO_2_ reactivity, CBFV and cerebral autoregulation seem to remain intact during sevoflurane administration or during propofol induced isoelectric electroencephalogram (EEG).

Although the central nervous system is the primary endpoint of most general anaesthetic it is still the least monitored organ in clinical anaesthesiology. In the last decade, technological research has expanded the application of near infrared spectroscopy to allow continuous, non-invasive and bedside monitoring of cerebral oxygen saturation providing accurate information on the balance between brain oxygen supply and demand.[1] Several clinical conditions routinely encountered in our daily medical practice have the potential to disrupt this balance exposing patients to the risk of intraoperative cerebral ischaemia. These alterations in brain oxygen balance remain undiagnosed if we do not use specific intraoperative cerebral function monitoring.

To date, monitors of cerebral function including electroencephalography, jugular bulb mixed venous oxygen saturation and transcranial doppler either require an invasive procedure or are not sensitive enough to effectively identify patients at risk for cerebral hypoxia. Near-infrared spectroscopy is a noninvasive device that uses infrared light, a technique similar to pulse oximetry, to penetrate living tissue and estimate brain tissue oxygenation by measuring the absorption of infrared light by tissue chromophores.[2] This technology can identify deficits in cerebral oxygenation, thus allowing for interventions or therapy to reverse cerebral oxygenation issues thereby preventing long term neurological sequelae.

The cerebrovascular system can undergo adaptive changes during laparoscopic surgery. Thus, it is important to anticipate increase in the CBFV after CO_2_ insufflation or any potential decrease in the oxygenation of the brain. By selecting the proper anaesthetic agent or by using specific surgical manoeuvres, possessing protective properties, regarding cerebrovascular dynamics can minimize the potential negative effects of pneumoperitoneum, hypercarbia and haemodynamic fluctuations on cerebrovascular balance.[3] Monitoring of rSO2 is a useful tool in clinical setting providing the ability to immediately detect alterations in cerebral perfusion, thereby giving the opportunity for early intervention. Due to the wide patient to patient variability of baseline rSO2 values, in each patient the baseline value should be determined before induction of anaesthesia as cerebral ischaemia is more related to the changes from baseline than to absolute values. A reduction of more than 20% from baseline is usually accepted as critical threshold of cerebral ischaemia. If baseline is lower than 50%, the critical threshold should be reduced to 15%.

Clearly, further studies are needed to enlighten the necessary measures for preventing possible alteration of CBFV and of brain oxygenation during specific operations in Trendelenbourg position, such as in laparoscopic surgery, especially in patients with cardiovascular and cerebral derangement. Nevertheless, routine use of rSO2 monitoring during laparoscopic surgery could actually guide anaesthesia and surgical plan in order to improve the patient outcome and shorten hospital stay. A simple noninvasive device as near infrared spectroscopy has the potential for optimizing the anaesthesiologists' and the surgical team's plans to the real needs of one of our main targeted organs: the brain.
